# Increased maternal TSH and decreased maternal FT4 are associated with a higher operative delivery rate in low-risk pregnancies: A prospective cohort study

**DOI:** 10.1186/s12884-015-0702-1

**Published:** 2015-10-16

**Authors:** L. Monen, VJ Pop, TH Hasaart, H. Wijnen, SG Oei, SM Kuppens

**Affiliations:** Department of Medical Health Psychology, Tilburg University Warandelaan, 2, 5037 AB, Tilburg, The Netherlands; Department of Obstetrics and Gynaecology, Catharina Hospital, Michelangelolaan 2, 5613 EJ Eindhoven, The Netherlands; Present: Department of Obstetrics and Gynaecology, Zuyderland MC, Henri Dunantstraat 5, 6419 PC Heerlen, The Netherlands; Midwifery Academy Maastricht, Universiteitssingel 60, 6229 ER Maastricht, The Netherlands; Department of Obstetrics and Gynaecology, Maxima Medical Centre Veldhoven, De Run 4600, 5504 DB Veldhoven, The Netherlands

**Keywords:** Maternal thyroid function, Failure to progress, Dystocia, Caesarean section, Operative delivery

## Abstract

**Background:**

The increasing number of operative deliveries is a topic of major concern in modern obstetrics. Maternal thyroid function is of known influence on many obstetric parameters. Our objective was to investigate a possible relation between maternal thyroid function, and operative deliveries. Secondary aim was to explore whether thyroid function was related to specific reasons for operative deliveries.

**Methods:**

In this prospective cohort study, low-risk Caucasian women, pregnant of a single cephalic fetus were included. Women with known auto-immune disease, a pre-labour Caesarean section, induction of labour, breech presentation or preterm delivery were excluded. In all trimesters of pregnancy the thyroid function was assessed. Differences in mean TSH and FT4 were assessed using *t*-test. Mean TSH and FT4 levels for operative deliveries were determined by one way ANOVA. Repeated measurement analyses were performed (ANOVA), adjusting for BMI, partiy, maternal age and gestational age at delivery.

**Results:**

In total 872 women were included, of which 699 (80.2 %) had a spontaneous delivery. At 36 weeks gestation women who had an operative delivery had a significantly higher mean TSH (1.63mIU/L versus 1.46mIU/L, *p* = 0.025) and lower mean FT4 (12.9pmol/L versus 13.3pmol/L, *p* = 0.007)) compared to women who had a spontaneous delivery. Mean TSH was significantly higher (*p* = 0.026) and mean FT4 significantly lower (*p* = 0.030) throughout pregnancy for women with an operative delivery due to failure to progress in second stage of labour, compared to women with a spontaneous delivery or operative delivery for other reasons.

**Conclusions:**

Increased TSH and decreased FT4 seem to be associated with more operative vaginal deliveries and Caesarean sections. After adjusting for several confounders the association remained for operative deliveries due to failure to progress in second stage of labour, possibly to be explained by less efficient uterine action.

## Background

Increasing rates of Caesarean Sections (CS) is a topic of major concern in obstetrics. Both the incidences of planned and emergency CS are rising, without necessarily better neonatal outcomes [1, 2]. Operative vaginal deliveries (OVD) have a slowly decreasing trend, but OVD are still common, especially in nulliparous women [1]. Maternal morbidity and mortality rates are higher for women with a CS or OVD [2, 3]. Many studies have been performed to determine risk factors associated with operative deliveries. The main risk factors for CS and OVD are nulliparity, induction of labour, increasing maternal age, abnormal position of the fetus, high maternal body mass index (BMI) and previous CS [4–6]. Most CS and OVD are performed due to failure to progress, especially in nulliparous women [6]. The main reason is inefficient uterine action and to a lesser extent cephalopelvic disproportion. In fact, efficient uterine action is more and more considered as the key to normal delivery [7, 8].

In previous studies it has been demonstrated that suboptimal maternal thyroid function (high maternal thyrotrophine stimulating hormone (TSH) and low free thyroxine (FT4)) is associated with adverse pregnancy outcomes [9–12]. Women with suboptimal thyroid function, mainly those with TPO-antibodies (thyroid-peroxidase-antibody), are at risk for miscarriage and preterm birth [9, 10]. Furthermore, there is evidence that high maternal TSH might interfere with normal obstetric outcome at term, with higher incidences of small for gestational age neonates [11], gestational diabetes [10] and meconium stained amniotic fluid [12]. Low FT4 has been associated with poor fetal neurodevelopment and psychomotor development in early childhood [13].

Little research is done into the relation between maternal thyroid function and uterine contractions. However, there is some evidence that suboptimal maternal thyroid function might be associated with more breech presentations, possibly due to increased stiffness of the myometrium [14]. This increased stiffness in patients with suboptimal thyroid function has been demonstrated in previous research in vascular smooth muscle cells as well [15, 16].

In the current study we evaluated a possible relationship between suboptimal maternal thyroid function and inefficient uterine action at term. Primary objective was to investigate a possible relation between maternal thyroid function and incidence of CS and OVD. Secondary objective was to explore whether thyroid function was related to specific reasons for operative deliveries: due to fetal distress or failure to progress. In order to create a representative low-risk group we excluded the women at high risk for operative deliveries (previous CS, inductions, breech presentations, multiple pregnancies).

## Methods

### Design and participants

Between January 1997 and April 1998, 1702 women who booked for antenatal visits at 12 weeks gestation were followed in five community midwife practices in the vicinity of Eindhoven (the Netherlands). Only Dutch Caucasian women were eligible to avoid language problems and confounding factors of ethnicity. General patient characteristics including BMI, education level, smoking status, maternal age and parity were collected. Information of possible previous pregnancies was collected. A cohort of women at low-risk for obstetric complications was defined, with the exclusion of preterm deliveries, breech presentations, multiple pregnancies and induction of labour. Women on thyroid medication, with type I diabetes and with a new diagnosis of overt hyper- or hypothyroidism at first screening were excluded. Delivery was considered an OVD when there was a ventouse or forceps delivery. In cases without CS or OVD, delivery was defined as spontaneous. The reasons for OVD and CS were classified as fetal distress, failure to progress in first stage of labour (prolonged dilatation) or failure to progress in second stage of labour (prolonged expulsion). Fetal distress was defined as a non-reassuring fetal heart rate with cardiotocography, as assessed by the responsible obstetrician using the FIGO classification.

**Fig. 1:**
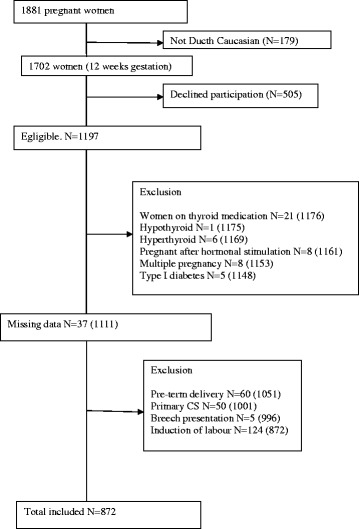
Selection process

The selection process is shown in Fig. 1 and the characteristics are shown in Table 1. This study was approved by the Medical Ethical Committee of Máxima Medical Centre in Eindhoven/Veldhoven. (METC project number: 0116) and written informed consent was obtained from all participants.

**Table 1:** Characteristics of a group of 872 women who delivered at term (≥37 weeks of gestation), comparing spontaneous deliveries and operative vaginal deliveries or secondary CS. Primary CS’s were excluded

	All deliveries (872)		Spontaneous deliveries (747)		Operative deliveries (125)		*p*-value
	Mean (SD) *N*(%)		Mean (SD) *N*(%)		Mean (SD) *N*(%)		*t*-test *X* ^2^
Age ≥35 yrs	30.5(3.6)		30.6 (3.7)		30.0 (3.4)		0.08
Low education		69 (8)		62 (8)		7 (6)	0.47
**BMI before pregnancy(kg/m2)**	**25.2(4.3)**		**25.0 (4.2)**		**26.3(4.7)**		**0.002**
**Primiparity**		**366(42)**		**268(36)**		**98 (78)**	**<0.001**
Miscarriage in obstetric history		164(19)		148(20)		16 (13)	0.06
Smoking		108(12)		89 (12)		19 (15)	0.30
*Thyroid function*							
*12 weeks gestation*							
TSH (mIU/L)	1.30 (2.97)		1.21(0.80)		1.88 (0.76)		0.33
FT4 (pmol/L)	16.2 (2.5)		16.2 (2.5)		16.2 (2.5)		0.98
*24 weeks gestation*							
TSH (mIU/L)	1.33 (0.69)		1.32(0.65)		1.45(0.89)		0.11
FT4 (pmol/L)	13.9 (2.0)		13.9 (2.0)		13.7 (1.8)		0.20
***36 weeks gestation***							
** TSH (mIU/L)**	**1.49 (0.75)**		**1.46(0.73)**		**1.63(0.86)**		**0.025**
** FT4 (pmol/L)**	**13.3 (2.2)**		**13.4 (2.2)**		**12.9 (2.1)**		**0.007**
TPO-Ab >35 IU/mL		75 (8.6)		60 (8.0)		15(12.0)	0.14
Family history thyroid dysfunction		158(18)		137(18)		21 (17)	0.80
*Neonatal outcome:*							
**Term at delivery (wks)**	**39.9(1.1)**		**39.8 (1.1)**		**40.3 (1.0)**		**<0.001**
Birth weight (gr)	3545(457)		3539(457)		3582(454)		0.33
Male offspring		451(52)		380(51)		71(57)	0.20

*Variables in bold are statistically significant (*P* < 0.05)

### Analysis

TSH was measured in serum at 12, 24 and 36 weeks using a solid-phase, two site chemiluminescent enzyme immunometric assay (IMMULITE Third generation TSH, Diagnostic Products Corporation, Los Angeles USA). The inter-assay coefficients of variation were 5.0 % and 4.4 % at concentrations 0.22 mIU/L and 2.9mIU/L, respectively. The non-pregnant reference range of TSH is 0.45 - 4.5 mIU/L. FT4 concentration was measured in serum at 12, 24 and 36 weeks with a solid-phase immunometric assay (IMMULITE Free T4). The inter-assay coefficients of variation for this technique were 6.7 % and 4.4 % at concentrations of 11.6 pmol/L and 31.5 pmol/L, respectively. TPO-Antibodies were determined in serum at 12, 24 and 36 weeks by means of the IMMULITE Anti-TPO-Ab kit. The inter-assay coefficients of variation for this analysis were 9 % and 9.5 % for concentrations of 40 kU/ml and 526 kU/ml, respectively. The anti-TPO assay is standardized in terms of the International Reference Preparation for anti-TPO MRC 66/387. Women were defined as TPO-Ab-negative when the titer was below 35 kU/ml at 12 weeks gestation. The highest TSH level found in our sample was 6.1 mIU/L. All measurements were performed in one laboratory.

### Statistical analysis

Statistical analysis was performed using the Statistical Package of Social Science (SPSS, 19.0). There were missing data in 37 cases, which were excluded from further analysis. The data from women who gave informed consent did not differ from the non-responders regarding maternal age, educational level and parity.

TSH and FT4 concentrations were not normally distributed. However, because the cohort size of all subgroups was substantial (n > 30) we were able to calculate differences in means (SD) of thyroid hormones concentration levels using Welch *T*-test (two-tailed). Differences in prevalence were calculated by chi-square. Subsequently, repeated measures ANOVA was performed to determine whether the changes of TSH and FT4 during pregnancy were associated with the mode of delivery and the different reasons for OVD or CS. We compared the changes of mean FT4 and TSH throughout pregnancy in women with and without a spontaneous delivery using repeated measurement analyses, adjusting for various selected confounders such as parity, gestational age, BMI and maternal age, as these are all of known influence on the risk of an operative delivery.

## Results

Table 1 shows that women who undergo an operative delivery have a higher pre-pregnancy BMI (26.3 kg/m^2^ versus 25.0 kg/m^2^) (*p* = 0.002) and have a longer gestational age (40.3 versus 39.8 weeks gestation) (*p* < 0.001). There were no statistically significant differences between a history of thyroid dysfunction in the family or the prevalence of positive TPO-antibodies. However, at 36 weeks gestation (third trimester) women who had an OVD or CS had a significantly higher mean TSH and lower mean FT4 compared to women who had a spontaneous delivery (TSH 1.63 mIU/L versus 1.46 mIU/L (*p* = 0.025) and FT4 12.9 pmol/L versus 13.4 pmol/L (*p* = 0.007) respectively). The 75 women with elevated TPO-Ab titres had significantly (*T*-test, two tailed, *P* < 0.001) higher mean TSH at 12, 24 and 36 weeks gestation compared to the remaining TPO-Ab negative women: 1.80 (SD: 1.21) versus 1.14 (0.68), 1.69 (SD0.98) versus 1.29 (0.60), 1.73 (SD:1.01) versus 1.46 (SD: 0.70), respectively. In the 797 TPO-Ab negative women at 12 weeks, we calculated the 2.5 and 97.5 percentile of TSH to define the normal reference range of TSH, this was 0.13 2.8 mIU/l. Subsequently we were able to assess the number of women with hypothyroxinemia at 12 weeks gestation: FT4 < 10^th^ percentile (< 13.4 pmol/l) with normal TSH values: 79 (9.1 %).

The different modes of delivery and the reasons for OVD and CS are shown in Table 2. 80.2 % of the women had a spontaneous delivery and in 19.8 % of the women an OVD or CS was performed. In 5.8 % of the deliveries there was an OVD or CS due to failure to progress in first stage of labour, in 7.9 % of the deliveries there was failure to progress in second stage of labour and 6.1 % of all deliveries were terminated due to fetal distress. Mean FT4 and mean TSH were compared between these subgroups using one way ANOVA. As shown in Table 2, mean TSH was significantly higher at 12 weeks of gestation (*p* = 0.010) and at showed a trend at 24 (*p* = 0.093) and 36 weeks gestation (*p* = 0.066) for women with an operative delivery due to failure to progress in second stage of labour. Mean FT4 was lower at a 90 % significance level at the first and third trimester of pregnancy for women with failure to progress in second stage of labour (*p* = 0.07, *p* = 0.16, p = 0.08 respectively).

**Table 2:** Mode of delivery in 872 women at term in whom labour started spontaneouslyANOVA analyses for influence of maternal thyroid function at every trimester on mode of delivery (df = 3)

	Spontaneous delivery	Failure to progress in 1^st^ stage of labour (prolonged dilatation)	Failure to progress in 2^nd^ stage of labour (prolonged expulsion)	Fetal distress	*P*
	*N* = 699 (80.2 %)	*N* = 51 (5.8 %)	*N* = 69 (7.9 %)	*N* = 53 (6.1 %)	
*12 weeks gestation*					
TSH(mIU/L) mean(SD)	1.20 (0.80)	1.21 (0.63)	2.01 (0.92)	1.21 (0.79)	0.010
FT4(pmol/L) mean(SD)	16.2 (2.75)	16.4 (1.98)	15.5 (2.60)	16.6 (2.23)	0.070
*24 weeks gestation*					
TSH(mIU/L) mean(SD)	1.31 (0.65)	1.38 (0.68)	1.53 (0.85)	1.35 (0.65)	0.093
FT4(pmol/L) mean(SD)	13.9 (2.0)	13.6 (1.6)	13.5 (1.7)	14.2 (2.0)	0.16
*36 weeks gestation*					
TSH(mIU/L) mean(SD)	1.46 (0.73)	1.48 (0.74)	1.71 (0.96)	1.57 (0.68)	0.066
FT4(pmol/L) mean(SD)	13.4 (1.9)	13.4 (2.2)	12.7 (1.9)	13.3 (2.1)	0.080

**Fig. 2:**
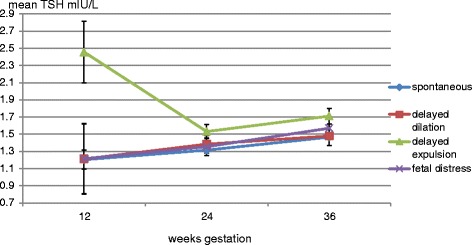
The relation between mean TSH at all trimesters and the mode of delivery in 872 term women in whom labour started spontaneously: women who had an operative vaginal delivery or Caesarean section because of prolonged expulsion had significantly higher mean TSH throughout gestation (repeated measures ANOVA, F = 3.1, *p* = 0.026.), adjusted for age, parity, gestational age and BMI

**Fig. 3:**
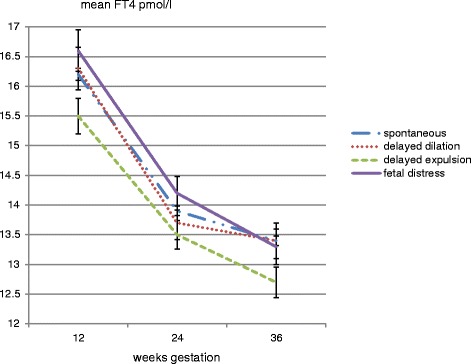
The relation between mean FT4 at three trimesters and the mode of delivery in 872 term women in whom labour started spontaneously: women who had an operative vaginal delivery or Caesarean section because of prolonged expulsion had significantly lower mean FT4 throughout gestation (repeated measures ANOVA, F = 3.0, *p* = 0.030), adjusted for age, parity, gestational age and BMI

As shown in Fig. 2, women who had an OVD or CS for failure to progress in the second stage of labour (delayed expulsion) had higher mean TSH levels (*p* = 0.026) at all trimester adjusted for age, parity, BMI and gestational age, compared to women who had a spontaneous delivery or an OVD or CS for other reasons. TSH was also significantly associated with parity. In Fig. 3 it is shown that women with an OVD or CS for failure to progress in second stage of labour, had a significant lower mean FT4 in all trimesters (*p* = .0.030), adjusted for age (*p* < 0.001), parity (*p* = 0.26), BMI (*p* < 0.001) and gestational age (0.44), compared to women with a spontaneous delivery or OVD or CS for other reasons (see Table 3). There were no differences in maternal thyroid function between women who had a spontaneous delivery compared to women who underwent an OVD or CS for fetal distress or failure to progress in the first stage of labour (delayed dilatation). When we repeated the analyses excluding women with hypothyroxinemia, similar results were found (data not shown).

**Table 3:** The relation between mean TSH and mean FT4 throughout gestation in term women in spontaneous labour, corrected for multiple counfounders

	F	*p*-value
Mean TSH		
Reason for operative delivery	3.107	0.026
Parity	5.005	0.026
Gestational age	0.037	0.85
Body Mass Index	0.007	0.93
Maternal age	0.716	0.40
Mean FT4		
Reason for operative delivery	3.006	0.030
Parity	1.276	0.26
Gestational age	0.605	0.44
Body Mass Index	16.469	<0.001
Maternal age	18.798	<0.001

## Discussion

In this study we found a significant association between high normal maternal TSH and low normal FT4 and the risk of operative delivery for women in spontaneous labour at term, not accounted for confounders. Our cohort consisted of 872 Caucasian women with a low-risk pregnancy. Planned Caesarean sections, inductions of labour, preterm deliveries and breech presentations were excluded. Our cohort was representative in terms of thyroid function and obstetric outcome. We did find a higher incidence of TPO-Ab in our study (8.6 %) compared to another large cohort study in the Netherlands (5.6 %) [17], but this can be explained by 40 % black women in their population, in whom TPO-Ab are less frequently found [18], while our sample included only white Caucasian women. Mean TSH and FT4 levels of the current study are comparable to their sample. Obstetric outcome was proportionate to a large cohort study carried out in the Netherlands to analyse trends in obstetric interventions[1]. They found a CS rate of 7.5 % for nulliparous women in spontaneous labour and an OVD rate of around 10 % between 1993 and 2002, with an increase in CS rates over the years [1]. Our total operative delivery rate was 20 %, for OVD and CS combined.

The current study showed that women who undergo a CS or OVD had a higher pre-pregnancy BMI and delivered at a later gestational age. This finding is consistent with previous reports [8, 19–21]. We also found that women with an operative delivery for failure to progress in second stage of labour had higher mean TSH levels and lower mean FT4 levels throughout pregnancy, compared to women who delivered spontaneously or had an operative delivery for another reason. This finding was adjusted for maternal age, pre-pregnancy BMI, parity and gestational age at time of delivery.

### Strength and limitations

One of the main strengths of this study is the fact that maternal thyroid function was assessed prospectively throughout pregnancy at fixed time-intervals. Repeated measurement analysis was performed to assess changes in thyroid function within and between subjects. Furthermore, we corrected for important confounders, including BMI, which is one of the known risk factors for operative deliveries [8, 19, 20]. We have not used cut off values for suboptimal maternal thyroid function. We believe that this is a major strength of this study, as there is considerable discussion about the proper cut off values and the definition of, for example, subclinical hypothyroidism in pregnancy [22, 23].

A limitation of the study is the fact that this was an observational study and therefore no conclusions on causality of the associations can be drawn. Furthermore, no strict definitions of fetal distress were used. Diagnosis of fetal distress was made on the basis of a non-reassuring fetal heart pattern. As only healthy Dutch Caucasian women were analysed in this study, extrapolation of the results to women with other ethnicities or to high risk pregnant women with for example hypertension or diabetes must be taken with caution. Furthermore, we did not consider iodine status in our cohort. However, it is known that in this region iodine intake is generally sufficient [24].

### Interpretation

Higher TSH and lower FT4 in this euthyroid sample were associated with higher operative delivery rates. However, TSH and FT4 were still in the normal ranges for all groups. In a large retrospective cohort study from Männistö et al. increased odds for CS (both prelabour and during labour) were found in women with primary hypothyroidism [25]. From that study however, it was not clear for what reasons the CS were performed.

The main difference in the current study, according to maternal TSH and FT4, is found in OVD and CS due to failure to progress in second stage of labour. It is known that the most important factor in the expulsion of the fetus is the thickness of the myometrium and thus the strength of the contractions, despite the pushing efforts of the mother [26]. It has not been well established whether the spontaneous contractions are stronger during the second stage of labour than during the first stage of labour, but it is known that during Valsalva the power of the contractions is significantly higher in the second stage of labour [27]. With Valsalva it is not only smooth muscle cell contractions, but contractions of the skeleton muscles of the abdominal wall as well, that determine the expulsive power. It is well known that thyroid hormones are of influence on the contractile phenotype of skeletal muscles [28]. The most effective uterine action is needed in the second stage of labour and factors that are of influence on the strength of the contractions will therefore become most apparent during this stage.

Calcium influx is necessary for excitation of the smooth muscle cells and therefore for the myometrial contractility required during labour. Parija et al. have demonstrated that hypothyroidism reduces calcium channel function in uterine tissue of the pregnant rat [29]. A study from Corriveau et al. has found that the amplitude and time course of contractions is enhanced in patients treated with thyroid hormone compared to controls [30]. This suggests that thyroid function is of direct influence on myometrial contractility. Besides the absolute strength of the contractions, it has also been found in vascular research that hypothyroidism leads to impaired smooth muscle cell relaxation, leading to increased artertial stiffness [15, 16]. Moreover, women with failed external cephalic version had higher TSH values, probably because of impaired uterine relaxation which is essential for the breech baby to turn [31]. The combination of the strength of the contractions and the impaired relaxation of the myometrium might lead to less efficient uterine action in women with suboptimal thyroid function in this study. Future research should confirm this finding in a larger population and determine whether treatment of suboptimal maternal thyroid function leads to less failure to progress. In our cohort the mean TSH and FT4 were all in the normal reference ranges for the pregnant population.

## Conclusions

Increased TSH and decreased FT4 seem to be associated with more operative vaginal deliveries and Caesarean sections. After adjusting for several confounders the association remained for operative deliveries due to failure to progress in second stage of labour, possibly to be explained by less efficient uterine action. The conclusions of this study are very relevant, as the increasing incidence of CS is one of the major concerns in modern obstetrics. Future research should confirm our findings and focus on determining the direct effect of maternal thyroid hormones on uterine contractions in vivo.

## Abbreviations

BMI: Body mass index
CS: Caesarean section
FT4: Free thyroxine
OVD: Operative vaginal delivery
TPO-Ab: Thyroid peroxidase antibodies
TSH: Thyrotropine stimulating hormone
